# Analysis of the consumption of sports supplements in elite fencers according to sex and competitive level

**DOI:** 10.1186/s13102-021-00278-0

**Published:** 2021-05-12

**Authors:** Fernando Mata, Raúl Domínguez, Álvaro López-Samanes, Ángela Sánchez-Gómez, Pablo Jodra, Antonio J. Sánchez-Oliver

**Affiliations:** 1 Centro de Estudios Avanzados en Nutrición (CEAN), Córdoba, Spain; 2 Departamento de Motricidad Humana y Rendimiento Deportivo, Faculty of Education Sciences, Universidad de Sevilla, Sevilla, Spain; 3 Studies Research Group in Neuromuscular Responses (GEPREN), University of Lavras, Lavras, Brazil; 4 Exercise Physiology Group, School of Physiotherapy, Faculty of Health Sciences, Universidad Francisco de Vitoria, Madrid, Spain; 5 Departamento de Enfermería Farmacología y Fisioterapia, Facultad de Medicina y Enfermería, Universidad de Córdoba, Córdoba, Spain; 6 Faculty of Education Sciences, University of Alcalá, Guadalajara, Spain

**Keywords:** Elite athletes, Ergogenic aids, Fencing, Sport nutrition, Sports performance

## Abstract

**Background:**

The aim of this study was to analyze the consumption of sports supplements (SS) in competitive level fencers and compare differences based on sex and competitive level (international and national).

**Methods:**

A total of 49 fencers (18 men and 31 women) of national (*n* = 16) and international (*n* = 33) level completed a questionnaire with questions about SS consumption and the possible repercussions on health and / or sports performance. The results were analyzed based on the different categorizations established by the Australian Institute of Sport (AIS), as well as by sex and level of competence to which the participants belonged to.

**Results:**

46.9% of fencers have consumed SS with the main motivation being performance improvement (34.2%). Medical doctors were the individuals who were more likely to advise men to consume SS (50.0% vs 5.6%; OR = 3.29 [1.50–7.20]). Friends were most likely to advise women (38.9% vs 8.3%; OR = 1.75 [1.05–2.93]). The most consumed SS were sport drinks (44.9%), vitamin C (43.4%), sport bars (38.8%), and caffeine (28.6%). In regards to the SS categories, it was observed differences in the interaction level·sex in medical supplements (*p* = 0.017). In addition, there was a higher prevalence of whey protein consumption in women (25.8% vs 0%; *p* = 0.020) and iron consumption in men (33% vs 6.5%; *p* = 0.039).

**Conclusions:**

The prevalence of SS use in fencers is within the values previously reported in athletes of the same competitive level. There were no differences by sex and competitive level in the total consumption of SS, nor in each of the groups of level of evidence, being sport drinks, bars and caffeine the most consumed SS.

## Background

Fencing was included as an Olympic sport for the first time in the 1896 Summer Olympics in Athens [1]. During a combat, fencers covered between 250 and 1,000 m [2]. This distance includes high intensity actions interspersed with submaximal actions that can cause neuromuscular fatigue, especially during competitions [3]. Thus, during competitions, mean oxygen consumption (VO_2_) is 53.9 and 39.6 ml/kg/min in male and female fencers respectively [4, 5]. As a result of these VO_2_ demands, a competitive fencer requires high conditioning of anaerobic and aerobic metabolism. In addition, fencing protective equipment increases the thermoregulatory demands on fencers, provoking an increase in the hydration demands [6, 7].

When an athlete’s performance level increases, their energy and nutrient intake becomes even more critical since any small benefit can provide a significant difference in competition results. In the Olympics, a difference corresponding to 1.6% can make the difference between finishing in first and fourth place [8]. These minimal differences encourage athletes to consider the consumption of sport supplements (SS) [9], which are defined as a food, nutritional component, or nutrient that is intentionally ingested. It is an addition to the usually consumed diet that has the aim of achieving certain physical performance or health benefits [10]. Despite this, only a small portion of current SS on the market have shown significant improvements in performance [11].

Different international institutions of reference publish positions stands periodically elaborated to establish the level of evidence behind different SS. Currently, three consensuses can be highlighted: first carried out approximately every 4 years by the Australian Institute of Sport (AIS) [12], second prepared by the group of experts on the International Olympic Committee (IOC) in 2018 [10], and the third from the International Association of Sports Nutrition (ISSN) [13]. In 2000, the AIS created the ABCD system whereby each SS is classified according to the level of scientific evidence. Those in group A are those with a high level of scientific evidence, which is subdivided into sports foods (i.e. sport bars, gels, sports drinks, or protein supplements), medical supplements (i.e. vitamin D or iron), and ergogenic aids (i.e. creatine, beta-alanine, bicarbonate, caffeine, or beetroot juice); supplements in group B are those that can have a positive effect in certain circumstances, but more evidence is still necessary; those in group C are those in which the evidence has concluded against their use; supplements in group D are prohibited substances [12]. Based on the criteria for SS safety, efficacy, and legality, the need arises to carry out a cost-benefit analysis before deciding to supplement athletes [11].

SS use is becoming more widespread, and current studies situate it between 30 and 95% [14]. Some of the most prominent variables of SS consumption include the type of physical activity, an athlete’s competitive level, and their sex. Among the most prominent variables of SS consumption type of physical-sporting activity that is carried out [15], level of competition of the athlete [16] or sex [9], being higher as it increases the competitive level of athletes and men (in the same sport) [9, 16]. No research has analyzed the prevalence and pattern of SS use in competitive level fencers. The demands of fencing necessitate athletes to improve their performance with an appropriate use of SS based on safety, effectiveness, and legality. The aim to this study is to analyze the consumption of SS in fencers based on their sex and competitive level (national vs international).

## Methods

### Experimental design

For data collection, fencers who voluntarily agreed to participate in this study completed a questionnaire about SS consumption. The administration of the questionnaire took place through the application of the online program, Google Forms, in which the athletes received the questionnaire on electronic devices specifically prepared for the study. At all times, the researchers were present to answer questions and direct the correct completion and delivery of the answers.

### Participants

A total of 49 fencers (18 men and 31 women, age: 21.8 ± 5.9 years) voluntarily participated in the study. All participants belong to the Royal Spanish Fencing Federation (RFEE) and compete in national events. Those selected by the national team compete in the world fencing circuit and compete at either the national (*n* = 16) or international (*n* = 33) level. Three researchers went to the RFEE national team technology center where fencers train and held an informative meeting to explain the characteristics of the study and obtain the informed consent of those volunteers who wanted to participate. The study is in accordance with the Declaration of Helsinki and was granted approval by the Ethics Committee of the Alfonso X El Sabio University.

### Instrument

The questionnaire was previously validated, evaluating the validity of its content, indicating its ability to measure what it was created for (a); its application, analyzing benefits and defects, reviewing the instructions of the instrument (b); its structure, reviewing the formulation of the questions, the proposed sequence and the response scale (c); and its presentation, in which the best characteristics of the instrument’s appearance and format were identified (d) [17]. This survey endorsed methodological quality in a meta-analysis, in which only 57 of the 164 questionnaires reviewed were approved [18]. In addition, the questionnaire has been used in different studies that have analyzed SS consumption in different sports modalities, exceeding 1000 athletes in total (from all the different studies) [9, 14, 16, 19–26]. The questionnaire is organized in three parts: (i) one in which the anthropometric, personal and social data of the respondent were collected, (ii) a second that aimed to analyze and contextualize sporting activity, and (iii) a final part aimed at relating the consumption of SS and its possible repercussions on health and / or sports performance. This final part included questions regarding who advised the athlete’s consumption of SS, the period of when they consumed SS (training period, competitive period, training and competitive period, period transition, always or never), and time relative to effort (before, during training, after training, or indistinctly).

### Statistical analysis

A Kolmogorov-Smirnov test was applied to contrast the normality of the variables and homoscedasticity using the Levene test. For the analysis of all quantitative variables that include descriptive aspects (height, weight, federated years, weekly training sessions) or the total number of SS consumed, as well as in each of the different established categories, an ANOVA of two factors (level and sex) was used, as well as the interaction of both factors. In the case of finding statistically significant differences, a Bonferroni Post-Hoc was applied. To analyze possible difference on the sex or competitive level of the fencers in relation to questions related to motivation expectations and contextualization, a chi-square test was applied being analyzed only SS that were consumed by at least 10% of the sample. The odds ratio (OR) was also calculated. Statistical significance was established at *p* < 0.05. Statistical analysis was performed using a statistical program SPSS (version 18.0 for Mac, SPSSTM).

## Results

Table 1 shows age (years), years of fencing experience, and weekly training sessions based on sex, level of competition, and the interaction of both sex and competitive level. As reported in the Table 1, no differences were observed by level of competition (*p* = 0.704), sex (*p* = 0.146), or the level · sex interaction (*p* = 0.840) as a function of age; unlike the years of fencing experience in which differences were observed for both sex (*p* = 0.008) and the level · sex interaction (*p* = 0.014). Thus, a greater training experience was observed in women with statistically significant differences in fencers at the national level (*p* = 0.002), but not in those at the international level (*p* = 0.866). In addition, in men, a greater difference was observed in those at the international level compared to those at the national level (*p* = 0.014). Regarding the weekly training sessions, although no differences were observed for sex (*p* = 0.460), international level fencers had a more significant tendency to conduct a greater number of training sessions (*p* = 0.064). finding differences statistically significant in women (*p* = 0.019).

**Table 1:** Characteristics of the sample according to sex and competitive level

Variable	Sex	International	National	Total	***p***-value Level	***p***-value Sex	***p***-value Level·Sex
Age (years)	Women	23.2 ± 8.2	22.1 ± 4.0	22.8 ± 7.0	0.704	0.146	0.840
	Men	20.0 ± 2.7	19.7 ± 1.4	19.9 ± 2.3			
	Total	22.0 ± 6.8	21.2 ± 3.4	21.8 ± 5.9			
Fencing (years of experience)	Women	9.5 ± 0.9	9.8 ± 0.4 ^a^	9.6 ± 0.9 ^a^	0.138	0.008	0.014
	Men	9.4 ± 0.9 ^b^	8.2 ± 1.5 ^a, b^	9.0 ± 1.2 ^a^			
	Total	9.5 ± 1.0	9.2 ± 1.2	9.4 ± 1.1			
Weekly training sessions	Women	6.8 ± 3.1 ^b^	4.4 ± 1.5 ^b^	6.0 ± 2.9	0.064	0.460	0.288
	Men	5.3 ± 1.3	4.7 ± 3.5	5.1 ± 2.2			
	Total	6.3 ± 2.7	4.5 ± 2.3	5.7 ± 2.7			

Data expressed as mean ± standard deviation. ^a^ statistically significant differences between women and men of the same performance level; ^b^ statistically significant differences between international and national level fencers of the same sex; Level of statistical significance was set as *p* < 0.05

When the fencers were asked if they favored the consumption of SS within the law regulation, 61.2% agreed, while 28.6% disagreed and 10.2% answered that they were unsure or did not answer, without observing statistically significant differences between sex (*p* = 0.457) or competitive level (*p* = 0.870). When asking participants if they had consumed SS in the past, 46.9% of the sample answered affirmatively. There were no statistically significant differences by sex (*p* = 0.390) or level of competition (*p* = 1.000).

Regarding the purpose or motivation for SS consumption, there were no observed differences by sex (*p* = 0.254) or level of competition (*p* = 0.346). The main reasons for SS consumption were improved performance (34.2%) followed by improved health (28.9%). Regarding the place of SS purchase, supermarkets (38.9%) and pharmacies (25%) were the most frequented. Similarly, no differences were detected by sex (*p* = 0.261) or competitive level (*p* = 0.349). The most frequent SS advisors were friends (26.7%), by doctors (23.3%), and coaches (20.0%). Although no differences were observed based on competitive level (*p* = 0.243), differences were observed based on sex (*p* = 0.016). Men were the most advised by doctors (50.0% vs 5.6%; OR = 3.29 [1.50–7.20]), family (16.7% vs 0%), and teammates (8.3% vs 5.6%; OR = 1.27 [0.28–5.84]). Friends (38.9% vs 8.3%; OR = 1.75 [1.05–2.93]), coaches (27.8% vs 8.3%; OR = 1.54 [0.90–2.63]), and the dietitian-nutritionist (22.2% vs 8.3%; OR = 1.43 [0.80–2.56]) were those that advised women on SS use.

Regarding SS time-of consumption, 14.3% of participants consumed SS only before exercise, 14.3% only during exercise and 14.3% only during exercise. Furthermore, 5.3% at breakfast; 2.9% at night; 17.1% at different timepoints throughout the day and 31.4% consumed it indifferently. There were no differences according to the level of competition (*p* = 0.699); however, there were differences by sex (*p* = 0.002). Thus, there was a higher proportion of men who consumed SS during sports practices (38.5% vs 0.0%; OR = 3.75 [2.03–6.94]), breakfast (15.4% vs 0.0%), and at night (7.7% vs 0.0%; OR = 2.83 [1.77–4.54]). Women more frequently consumed SS in different doses throughout the day (27.3% vs 0.0%), after exercise (22.7% vs 0.0%), and before exercise (18.2% vs 7.7%; OR = 1.33 [0 .77–2.30]). Of note, the percentage of male and female fencers who took SS indifferently was similar (30.8% vs 31.8%, respectively; OR = 0.97 [0.37–2.56]).

In relation to the total number of SS consumed, similar patterns were observed in men and women (3.28 ± 3.03 vs 3.35 ± 3.73; *p* = 0.562) along with international and national fencers (3.12 ± 2.81 vs 3.75 ± 4.58; *p* = 0.848) (Table 2). Similarly, no statistically significant differences were observed in the total number of SS consumed in Groups A, B, and C of the AIS, regardless of sex, level of competition, or level·sex (*p* > 0.05). Regarding the SS subgroups in group A, there were no differences based on the level of competition, sex, and the interaction level·sex (*p* < 0.05) in sports foods and ergogenic aids. Differences within the medical supplements subgroup were observed: international-level fencers had higher SS consumption (*p* = 0.060), with differences in the interaction level·sex (*p* = 0.017)*.* International-level male fencers had statistically higher SS consumption (*p* = 0.008) while women did not (*p* = 0.654). as well as differences in men of international level (*p* = 0.001) (see Table 2).

**Table 2:** Number of supplements consumed depending on sex and competitive level according to AIS categories (14)

AIS Categories		Sex	International	National	Total	***p***-value Level	***p***-value Sex	***p***-value Level·Sex
**Total**		Women	2.76 ± 2.79	4.60 ± 5.15	3.35 ± 3.73	0.848	0.562	0.144
		Men	3.75 ± 2.86	2.33 ± 3.39	3.28 ± 3.03			
		Total	3.12 ± 2.81	3.75 ± 4.58	3.33 ± 3.45			
**Gruop A**	Sport Foods	Women	0.90 ± 1.22	1.40 ± 1.27	1.06 ± 1.24	0.901	0.583	0.132
		Men	1.25 ± 0.87	0.67 ± 0.82	1.06 ± 0.87			
		Total	1.03 ± 1.10	1.13 ± 1.15	1.06 ± 1.11			
	Medical Spplements	Women	0.29 ± 0.64 ^a^	0.40 ± 0.70	0.32 ± 0.65	0.060	0.582	0.017
		Men	0.92 ± 0.79 ^a, b^	–	0.61 ± 0.78			
		Total	0.52 ± 0.76	0.25 ± 0.58	0.43 ± 0.71			
	Ergogenic aids	Women	0.38 ± 0.70	0.50 ± 0.53	0.42 ± 0.62	0.604	0.447	0.927
		Men	0.25 ± 0.45	0.33 ± 0.82	0.28 ± 0.57			
		Total	0.33 ± 0.59	0.44 ± 0.63	0.37 ± 0.60			
	Total	Women	1.57 ± 1.66	2.30 ± 2.11	2.42 ± 1.51	0.528	0.676	0.053
		Men	2.42 ± 1.51	1.00 ± 1.55	1.94 ± 1.63			
		Total	1.94 ± 1.63	1.81 ± 1.97	1.86 ± 1.73			
**Group B**	Total	Women	0.48 ± 0.87	1.00 ± 1.15	0.65 ± 0.98	0.343	0.723	0.491
		Men	0.58 ± 1.08	0.67 ± 1.03	0.61 ± 1.04			
		Total	0.52 ± 0.94	0.88 ± 1.09	0.63 ± 0.99			
**Group C**	Total	Women	0.71 ± 0.90	1.30 ± 2.21	0.90 ± 1.45	0.563	0.491	0.441
		Men	0.75 ± 1.22	0.67 ± 1.21	0.72 ± 1.18			
		Total	0.73 ± 1.00	1.06 ± 1.88	0.84 ± 1.34			

Data expressed as mean ± standard deviation. ^a^ statistically significant differences between women and men of the same performance level; ^b^ statistically significant differences between international and national level fencers of the same sex; Level of statistical significance was set as *p* < 0.05

Table 3 shows the SS most consumed by the sample (9 SS that presented a consumption rate higher than 10%). The most consumed SS were sport drinks (44.9%), bars (38.8%), and caffeine (28.6%). When comparing the frequency of consumption according to sex, differences were observed in the consumption of whey protein—which was more frequently consumed in women (*p* = 0.020)—and iron in which women had lower iron consumption (*p* = 0.039; OR = 0.39 [0.21–0.73]). With respect to competitive level and SS consumption frequency, international-level fencers had a higher statistical difference of iron consumption (*p* = 0.041).

**Table 3:** Most commonly used supplements according to sex and competitive level according to AIS categories (14)

AIS categories		Supplements	Sex					Competitive level			
			Total	Female	Male	***P***-value	OR	International	National	***P***-value	OR
**Group A**	Sports Foods	Sports drinks	44.9%	35.5%	61.1%	0.136	0.52 [0.24–1.11]	45.5%	43.8%	1.00	1.05 [0.47–2.36]
		Sports Bars	38.8%	35.5%	44.4%	0.559	0.79 [0.38–1.64]	39.4%	37.5%	1.00	1.06 [0.46–2.43]
		Whey protein	16.3%	25.8%	–	0.020 *	–	15.2%	18.8%	1.00	0.85 [0.31–2.30]
	Medical supplements	Iron	16.3%	6.5%	33.3%	0.039 *	0.39 [0.21–0.73]	24.2%	–	0.041 *	–
		Vitamin D	12.2%	12.9%	11.1%	1.00	1.12 [0.34–3.69]	12.1%	12.5%	1.00	0.98 [0.29–3.28]
	Ergogenic aids	Caffeine	28.6%	32.3%	22.2%	0.527	1.40 [0.56–3.52]	27.3%	31.3%	1.00	0.88 [0.37–2.07]
**Group B**		Vitamin C	20.4%	16.1%	27.8%	0.465	0.67 [0.31–1.43]	15.2%	31.3%	0.261	0.56 [0.25–1.25]
		BCAA	10.2%	16.1%	–	0.143	–	9.1%	12.5%	1.00	0.79 [0.25–2.53]
**Group C**		Green tea	18.4%	19.4%	16.7%	1.00	1.13 [0.41–3.08]	18.2%	18.8%	1.00	0.98 [0.40–2.72]

*SS* Sports supplements; * statistically significant differences between women and men of the same performance level (*p* < 0.05). *BCAA* Branched chain amino-acids

## Discussion

The aim of this study was to determine the prevalence of SS use in elite fencers with respect to sex and competitive level (national vs international). Although there are few studies in different contexts (i.e., training and sports nutrition fields) in elite fencers [3–5, 7], this is the first study that specifically analyzes the consumption of SS in fencing.

The proportion of SS consumption differs greatly depending on a multitude of variables, such as the sports modality that has the greatest difference between them (30–95%) [14, 18]. Although our study’s results place SS consumption within this range (46.9%), this was lower than similar populations from other sports modalities that reported consumption between 64 and 81.7% [16, 19, 27, 28]. This places SS consumption within our sample of elite fencers below the findings of recent studies that have evaluated the consumption of SS in elite athletes.

In addition to the sport modality, two variables that most influence the consumption of SS are the level of competition and sex [10]. The current hypothesis is that fencers at a higher level of competition [18] who are male [29] consume more SS. However, the results obtained in our study do not show a significant difference between national and international fencers, which contrasts with studies in which there are significant differences international-level athletes [9, 16, 18, 21, 27]. Similarly, no differences were observed in the prevalence of SS use among athletes according to sex, which is in line with the findings of the meta-analysis by Knapik et al. (2016) [18]. These data contrast with those currently found in elite Spanish athletes in which a higher consumption was observed in men vs women, 67% vs 58% (27) and 54.8% vs 45.5% [9].

As previously stated, our data agrees with the principal reason for SS consumption in competitive fencers: performance improvement; however, the proportion of fencers who report this motivation (34.2%) is lower than that reported in athletes of other sports modalities (45–77.8%) [9, 16, 21, 28].

The most frequent places of SS purchase were supermarkets (38.9%) and pharmacies (25%). It should be added that the data contrast with those obtained in a similar population that primarily attended specialty stores (45%) or sought sponsorships and the internet (25%) [30]. Although there is similarity in terms of the data obtained from purchasing in pharmacies in dinghy sailors [9], supermarkets are not the usual place of SS purchase in currently published studies [9, 14, 16, 22].

Bearing in mind that the possible effects of poor SS selection can lead to unintentional doping and/or health risks, the sources of information in which athletes rely on for SS consumption are important [31]. Although dietitian-nutritionists or sports doctors are the most ideal expert sources of SS consumption [30], the most frequent advisors of SS consumption were friends (26.7%), doctors (23.3%), and coaches (20.0%). These data are similar to those reported in studies carried out in young athletes of different sports modalities [9, 32] or in amateur adult athletes [19, 23]. Our study demonstrates the need to influence the correct choice of sources of advice on SS, although in this case, the sample presents a low consumption of SS from group C according to the AIS [12]. Male fencers were more likely to seek advice from doctors (*p* = 0.016)—a question that may be related to their greater intake of iron medical supplements (*p* = 0.039).

The moment of SS ingestion plays an important role in optimizing performance and recovery [33]. No differences were observed between levels of competition; however, significant differences were observed based on sex (*p* = 0.002), establishing that men preferred intake SS during sports activity while women throughout the whole day.

Although the data obtained in relation to the SS ingestion moment were in general and not in a specific way for each of the SS, the fact that only 14.3% of the athletes in the sample consumed SS prior to the effort has a practical implication, since, the consumption of an SS consumed by 28.6% of the sample, such as caffeine, should be ingested 45–60 min before the effort [10, 34].

The mean number of SS consumed by the sample is lower than that reported in a recent study of sailors (3.9 SS) [9], squash players (8.4 SS) [16], and other modalities such as bodybuilding in which they reported mean intakes of 20 SS daily [14]. The total intake of SS did not differ according to sex or level of competition, which run contrary to the hypothesis that sex and level of competition influence SS consumption [10, 18]. Similarly, statistically significant differences of total SS consumed were not observed by sex or level of competition in Groups A, B, and C [12]. These results are similar to those obtained from squash players where no significant differences were observed between the consumption of supplements in groups A, B and C when comparing athletes of international vs national level [16]. These results, however, run contrary to that of elite sailors where there was a higher consumption of Groups A/B SS in international-level sailors and Group C SS in national-level sailors [9]. According to the results obtained in our study, there were only differences in the group of medical supplements (under group A) when comparing athletes by sex: men reported higher SS consumption. This is similar to results found in another study where there were only differences in sport foods by competitive level [9].

When comparing the SS most consumed by the sample according to sex, a higher consumption of whey proteins was observed in women when compared to men that reported no consumption (25.8% vs 0%; *p* = 0.020). This contrasts with a study carried out in 504 Spanish elite athletes of different modalities in which men had higher protein supplementation (50.9% vs 29%; *p* < 0.01) [35]. Among other reasons, this can be explained by a greater need and / or concern to improve strength and muscle mass through the use of supplements in male athletes [14, 36–38]. In addition to protein supplementation, a significant difference was observed in terms of iron consumption: women reported lower consumption (6.5% vs 33.3%; *p* = 0.039; OR = 0.39 [0.21–0.73]), contrary to those reported in the aforementioned study by Aguilar-Navarro et al. (2020) (22% vs 11%; *p* = 0.01) [35]. A possible explanation for lower iron consumption amongst our study’s participants is that unlike male fencers, female fencers are less frequently recommended iron supplementation by doctors who might recommend it for prophylactic purposes in men. This could lead to health and performance problems for women since the prevalence of iron deficiency is higher in female athletes [38], because of higher iron demands from menstruation [37]. Furthermore, both findings are contrary to what was reported in a meta-analysis carried out by Knapik et al. (2016) in which iron supplementation was higher in women and protein supplementation (including creatine) was higher in men [18].

When comparing the SS most consumed by the sample according to competitive level (international vs national), the only differences were observed in iron consumption, which was greater in international-level athletes (24.2% vs 0%; *p* = 0.04). These results differ from those reported in elite Spanish sailors that had no differences in SS use [9]. However, our results are similar to those found in Spanish squash players that had higher SS consumption at the international level without statistical significance (35.7% vs 14.3%; *p* = 0.11). The higher consumption in international-level athletes could be explained by iron being an essential component of myoglobin and hemoglobin, which ensure adequate oxygen supply to skeletal muscles [39]. A nutritional deficiency may compromise energy metabolism, thereby increasing glycolysis while reducing energy efficiency, performance, and training adaptations [36].

An important finding of the present study is that the 3 most consumed SS by the entire sample were sports drinks, sports bars and caffeine. According to sex, sport drinks, sports bars and iron were the most consumed by men while sports drinks, sports bars and caffeine by women respectively. In addition, according to competition level at international level consumed sports drinks, sports bars and caffeine, while at national level reported sports drinks, sports bars and caffeine/vitamin C (same consumption level in this category). These findings are in line with the data reported in other studies in which SS are some of the most consumed substances by elite athletes of various sports modalities [9, 16, 28].

In fencing, fencers are equipped with protective clothing that increase body temperature, the sweating rate, and thus, fluid and electrolyte losses [7]. The high consumption of sport drinks (44.9%) in our sample may be due to these characteristics. Thus, an inadequate fluid-balance during training or competitions could provoked a loss of performance (i.e., endurance, muscle power, attention, or speed in decision making actions) [6, 7]. Sports drinks may not only help to maintain fluid balances, but also retain carbohydrates that maintain carbohydrate oxidation and blood glucose levels while reducing the use of muscle and liver glycogen stores. Added together, sport drinks can contribute to improvements in performance. Although it is to be noted that this may depend on environmental conditions, training intensity, and competition duration [40]. In addition, it should be underscored that the consumption of sport drinks was higher in men than in women (61.1% vs 35.5%). Thus, this could be due to a higher rate of sweating in [6, 41]. which would be aggravated by the characteristics previously mentioned in this sport modality [7].

Sport bars were the second most consumed SS by the sample (38.8%) with no differences in performance level or sex. These results are similar to those observed in other types of sports [9, 16]. This may be due to the usefulness of this supplement in international trips and competitions since they are an easily accessible resource for consumption and nutrient delivery [10, 11] on the one hand, and can serve in this type of athletes before, during and then in more demanding training and competitions compared to nationals [42].

Caffeine was the most used SS within the AIS Group A ergogenic aid subgroup (28.6%). Although there were no significant differences by competitive level and sex, its consumption was somewhat higher in national-level (31.3% vs. 27.3%) and women (32.3% vs 22.2%) fencers. These data are similar to those found in certain studies [16, 18], but contrary to others [9, 21]. Caffeine is one of the few SS studied in fencing [43, 44]. In a study by Bottoms et al. (2013) [44] conducted in professional fencers of both sexes, it was found that supplementation with 3 mg/kg of caffeine had no effect on the information processing speed measured by a Stroop test the ability on a lungest test or hit a target, although the ratings of perceived exertion (RPE) decreased. Doyle et al. (2016) [43] analyzed the effect of different doses of caffeine (from 1.5 to 7.5 mg/kg) on arousal, response time, and accuracy during a simulated fencing practice in competitive-level fencers. Caffeine supplementation increased arousal in a dose-dependent relationship, although an ergogenic effect was only observed from supplementation with doses of 4.5–6 mg / kg. Thus, it was found that the increase in psychological tension levels within an optimal range is related to improved performance [45]. Given that caffeine has a chemical form similar to adenosine by blocking adenosine receptors, this supplementation affects neuromuscular performance, mood, and subjective perception of effort [46, 47], allowing for a fencer to achieve an optimal state of mood and reduce RPE [43, 44].

In addition to the previous SS, fencers can improve their sports performance with other ergogenic aids from Evidence Group A (AIS 2019) that had 0% prevalence of consumption in the present sample, such as B-alanine (due to its ability to regulate pH at the intracellular level) [48–51], sodium bicarbonate (due to improvement in extracellular pH regulation) [52, 53], and / or beetroot juice (capable of improving contractility and muscle power) [53, 54].

Regarding the SS of group B (AIS, 2019) [12], the consumption of vitamin C (20.4%) stood out in men (27.8% vs 16.1%) and national fencers (31.3% vs 15.2%). These data are below comparing meta-analysis by Knapik et al. (2016) [18] that placed their consumption at around 32%, but similar to those reported in Dutch athletes of different disciplines, which placed it at around 23% [32]. However it is worth to mentioned that vitamin C consumption was higher in international squash players (35.7% vs 25%) although it was not significant [16]. Regarding the SS of group C (AIS, 2019) [12], it is worth highlighting the consumption of green tea for the total sample (18.4%), regardless of sex and level of competition (16.7–19.4%). Green tea use is typically more common in recreational athletes who usually use it for aesthetic purposes (i.e., fat loss) despite no evidence for its use existing [17, 22], especially within elite athletes [9, 16, 21, 28]. This may be due to the fact that the most frequent advisor in the present sample were friends, which could lead to the consumption of a supplement without any level of evidence.

### Practical implications

Although the use of supplements is widespread among athletes, it is important that athletes and nutrition specialists who care for them perform a cost-benefit analysis on their appropriate and responsible use [11, 55]. This analysis must be developed based on the prevalence of SS use in fencers, which is within the values previously reported in athletes of the same competitive level. There were no significant differences by neither sex nor level of competition in the purpose of consumption and the place of SS purchase. Likewise, there were no differences by sex and competitive level in the total consumption of SS, nor in each of the groups of level of evidence. The SS most consumed were sport drinks, bars, and caffeine, presenting differences in the consumption of whey protein by sex and in iron by interaction (sex and level) of competition. These findings highlight the importance of a complete nutritional assessment, as well as the individual context and needs of the athlete at that time [56]. Importantly, the use of SS should not compensate poor food choices and an inadequate diet with the exception of being a short-term supplement when dietary changes are not possible [11]. Also, a well-chosen diet supports the benefits of using evidence-based supplements, whether taken to maximize performance, delay fatigue, alter body composition, or improve health [57], and that any recommendation for athletes should be based on current scientific data and individual needs.

## Conclusions

The prevalence of the use of SS in fencers is within the range previously reported in athletes of the same competitive level. There were no significant differences neither by sex nor by level of competition in the purpose of consumption and the place of SS purchase. If there were significant differences in the role of sex in the counselor and the time of consumption of SS. Likewise, there were no differences by sex and competitive level in the total consumption of SS, nor in each of the groups of level of evidence. The SS most consumed were sport drinks, bars, and caffeine. Finally, differences were reported in the consumption of whey protein by sex while iron presented statistical differences by sex and level of competition.

## Data Availability

The datasets used and/or analyzed during the current study are available from the corresponding author on reasonable request.

## Abbreviations

AIS: Australian Institute of Sport
IOC: International Olympic Committee
ISSN: International Association of Sports Nutrition
RPE: Ratings of perceived exertion
RFEE: Royal Spanish Fencing Federation
SS: Sport supplements
VO_2_: Oxygen consumption
